# Host range of *Microsporidia MB* in aquatic macrofauna inhabiting *Anopheles* breeding sites

**DOI:** 10.1128/spectrum.00679-26

**Published:** 2026-09-02

**Authors:** Cynthia Nyambura King’ori, Collins Odongo Okoth, Joel Ltilitan Bargul, Sevgan Subramanian, Jeremy Keith Herren, Thomas Ogao Onchuru

**Affiliations:** 1 International Centre of Insect Physiology and Ecology (ICIPE), Kasarani, Nairobi, Kenya; 2 Department of Biochemistry, Jomo Kenyatta University of Agriculture and Technology 118985, Nairobi, Kenya; 3 Department of Physical and Biological Sciences, Bomet University 530383, Bomet, Kenya; Connecticut Agricultural Experiment Station, New Haven, Connecticut, USA

**Keywords:** *Microsporidia MB*, malaria, *Plasmodium*, endosymbionts, *Anopheles*

## Abstract

*Microsporidia MB*, a recently discovered endosymbiont *Anopheles arabiensis* mosquitoes, shows great potential as a malaria-blocking tool due to its *Plasmodium*-blocking phenotype. The symbiont utilizes vertical (mother-to-offspring) and horizontal (sexual) transmission routes to propagate across anopheline populations. *Microsporidia MB* occurs throughout the *Anopheles* life cycle, with no evidence to date of the existence of a secondary host. In this study, we conducted a comprehensive molecular screening of *Microsporidia MB* in aquatic macrofauna associated with *Anopheles* larval habitats to determine whether they serve as secondary hosts for *Microsporidia MB*. We collected aquatic macrofauna from anopheline breeding sites along the Ahero irrigation scheme, including insects from the orders Hemiptera (families Naucoridae, Micronectidae, Nepidae, and Notonectidae), Coleoptera (families Staphylinidae, Noteridae, Hydrophilidae, and Dytiscidae), Diptera (anopheline and non-anopheline larvae and pupae), Orthoptera, and Ephemeroptera; snails (Hygrophila); crustaceans (Podocopida); and frogs (Anura). We detected *Microsporidia MB* in 15.04% of anopheline larvae, but not in non-anopheline mosquito larvae, pupae, or any other aquatic macrofauna, despite their continuous interaction with anopheline mosquitoes in the larval habitats. The absence of *Microsporidia MB* detection across the diverse assemblage of aquatic macrofauna surveyed in this study suggests that these taxa are unlikely to constitute major secondary hosts or reservoirs of the symbiont. While broader surveys across additional taxa are needed to fully exclude alternative hosts, the consistent detection of *Microsporidia MB* at high infection intensities in *Anopheles* mosquitoes supports a strong host association. This reinforces its potential as a targeted, species-specific, and environmentally safe symbiont-based intervention for malaria control.

**IMPORTANCE:**

Malaria continues to pose a significant global health burden, particularly in sub-Saharan Africa, and causes thousands of deaths annually. *Microsporidia MB* is an anopheline-associated symbiont that is vertically and horizontally transmitted in *Anopheles* mosquitoes and shows great promise as a complementary malaria control tool due to its *Plasmodium* blocking phenotype. However, its lifecycle is not fully understood. Understanding its lifecycle and host range is important for assessing the symbiont’s ecological safety and feasibility for field release. This study found no evidence of *Microsporidia MB* in a wide range of aquatic macrofauna using sensitive molecular screening, despite their frequent contact with infected mosquito larvae. While non-detection does not rule out rare or transient infections, the results suggest that these organisms are unlikely to serve as major hosts of the symbiont. This apparent host specificity reduces concerns about spreading to non-target species and supports the development of *Microsporidia MB* as a targeted malaria-control intervention.

## INTRODUCTION

Malaria is an endemic disease that remains a significant global health burden, with the greatest effect experienced in sub-Saharan Africa. In 2024, there were an estimated 282 million malaria cases globally from 80 endemic countries, and 610,000 global malaria deaths (1). Africa shouldered the greatest burden of the disease, accounting for 94% of these reported cases, and five African countries accounted for almost half of the reported cases globally (1). The current malaria prevention tools heavily rely on the use of insecticide-treated bed nets, rapid disease detection, residual spraying (2), and more recently, the endorsement of two malaria vaccines (RTS,S/AS01 and R21/Matrix-M) by the WHO.

However, these tools face significant challenges in their effectiveness. While the RTS,S/AS01 and R21/Matrix-M have demonstrated modest efficacy in reducing malaria cases (33–36% over 4 years and 75% over 18 months, respectively), the vaccines require multiple-dose administration (3, 4). This requirement creates a bottleneck for at-risk communities in rural areas who may not be able to access health centers frequently, limiting the effectiveness of the vaccine (4). Additionally, the cold chain requirements for vaccine delivery may not be reliably achieved in rural areas, limiting the effectiveness of the vaccine (4). Also, the deletion of *Pfhrp2* and *Pfhrp3* genes in *Plasmodium falciparum* renders these parasites incapable of expressing the histidine-rich protein, the primary target antigen for malaria rapid diagnostic tests, leading to false negative results (2). This affects the accuracy of the current tests used in malaria detection. Similarly, increased resistance of *Anopheles* mosquitoes to pyrethroids and DDT in treated bed nets and changes in vector biting behavior have reduced the effectiveness of the treated bed nets and residual spraying (5, 6). Additionally, insecticide-treated bed nets and residual spraying are not efficient intervention methods where the entomological inoculation rate (EIR) is higher than 1,000 infectious bites per person per year (7). This has necessitated new malaria control initiatives to complement existing tools to reduce the burden of malaria.

One promising strategy is the use of naturally occurring symbionts as control measures for vector-borne diseases (VBDs). Extensive research has been conducted, for instance, on the use of *Wolbachia* as a possible biocontrol of VBDs. *Wolbachia* is a naturally occurring and maternally transmitted endosymbiont in mosquitoes that is being used as a biocontrol tool for dengue virus (DENV) (8). This is possible because it elicits pathogen interference (PI) phenotype in *Aedes aegypti* mosquitoes by reducing their susceptibility to a variety of dengue virus (DENV) isolates and decreasing viral titers in the body and saliva of the few infected mosquitoes (9). Efforts to identify similar endosymbionts for malaria control are underway, with the discovery of several microbes, including *Microsporidia MB*, that efficiently inhibit *P. falciparum* transmission (10).

*Microsporidia MB* is an anopheline-associated symbiont that is vertically transmitted from the mother to offspring at 45–100% efficiency (10). It is also horizontally transmitted through mating at a relatively higher frequency from males to females (41.5–56%) than from females to males (22.4–33%) (11, 12). Recent studies have demonstrated that *Microsporidia MB*-infected males exhibit approximately twofold higher mating competitiveness than their uninfected counterparts (12), a trait that may further enhance the transmission and persistence of the symbiont in natural mosquito populations. This symbiont interferes with the capacity of *Anopheles arabiensis* to transmit the *Plasmodium* parasite by inhibiting the development of oocysts into transmissible stages (10). The *Plasmodium* transmission-blocking ability of *Microsporidia MB*, its vertical and horizontal spread within vector populations, and its relative fitness advantage to the vector make it a promising candidate for developing a symbiont-based malaria control strategy.

Elucidating the life cycle of *Microsporidia MB* is fundamental to designing effective deployment strategies for its use in malaria control programs. Previous studies have shown that *Microsporidia MB* infects the germline of *An. arabiensis* ovaries, from where it establishes in the developing eggs and is subsequently vertically transmitted to the next generation (13, 14). Further, the symbiont undergoes sporogony in some of the developing oocytes, resulting in the formation of eggs that are packed with *Microsporidia MB* spores (15). The role of these spores in *Microsporidia MB* transmission is still unknown. In most microsporidian species, spores are the dormant, environmentally resistant, and infective form of the microbe and are commonly produced to infect susceptible hosts (16). In some microsporidian species, such as *Amblyospora*, *Hyalinicysta*, and *Parathelohania* spp., spores produced by the primary host infect an intermediate host where the microsporidian microbe further develops before completing its lifecycle in the primary host (17, 18). The infective capacity of *Microsporidia MB* spores found in oviposited eggs is still under investigation. So far, *Microsporidia MB* has only been reported in anopheline mosquitoes, where it is maintained through vertical (mother-to-offspring) and horizontal (sexual) transmission (10, 11, 13, 14, 19–21), and it is unclear if it has a secondary host.

Anopheline mosquitoes oviposit and develop as larvae and pupae in aquatic habitats ranging from small temporary pools to large permanent water bodies that they share with other aquatic organisms (22). Members of Odonata (e.g., dragonfly and damselfly nymphs), Coleoptera (e.g., Dytiscidae), Hemiptera (e.g., Notonectidae, Belostomatidae), Diptera (e.g., Toxorhynchites), and Crustacea (e.g., copepods) not only share aquatic habitats with the anopheline mosquitoes, but also prey on aquatic stages of these mosquitoes, hence their application in controlling mosquito populations (7, 23). Because these predators co-exist with aquatic stages of anopheline mosquitoes in larval habitats and prey on them, they are likely to encounter and consume eggs containing *Microsporidia MB* spores, thereby providing a potential route for cross-species exposure or infection. While prior studies screened crustaceans, Culicoides midges, and other mosquito species found in anopheline larval habitats and found no *Microsporidia MB* infection in non-anopheline species (11), other common mosquito larval predators and aquatic macrofauna often found in larval habitats were not investigated. This study aimed to determine whether mosquito predators and other aquatic macrofauna commonly found in anopheline larval habitats serve as a secondary host for *Microsporidia MB*. By improving our understanding of *Microsporidia MB* ecology, transmission dynamics, and life cycle, this study lays critical groundwork for the future development and implementation of *Microsporidia MB* as a sustainable malaria control tool.

## MATERIALS AND METHODS

### Collection site

**Fig 1:**
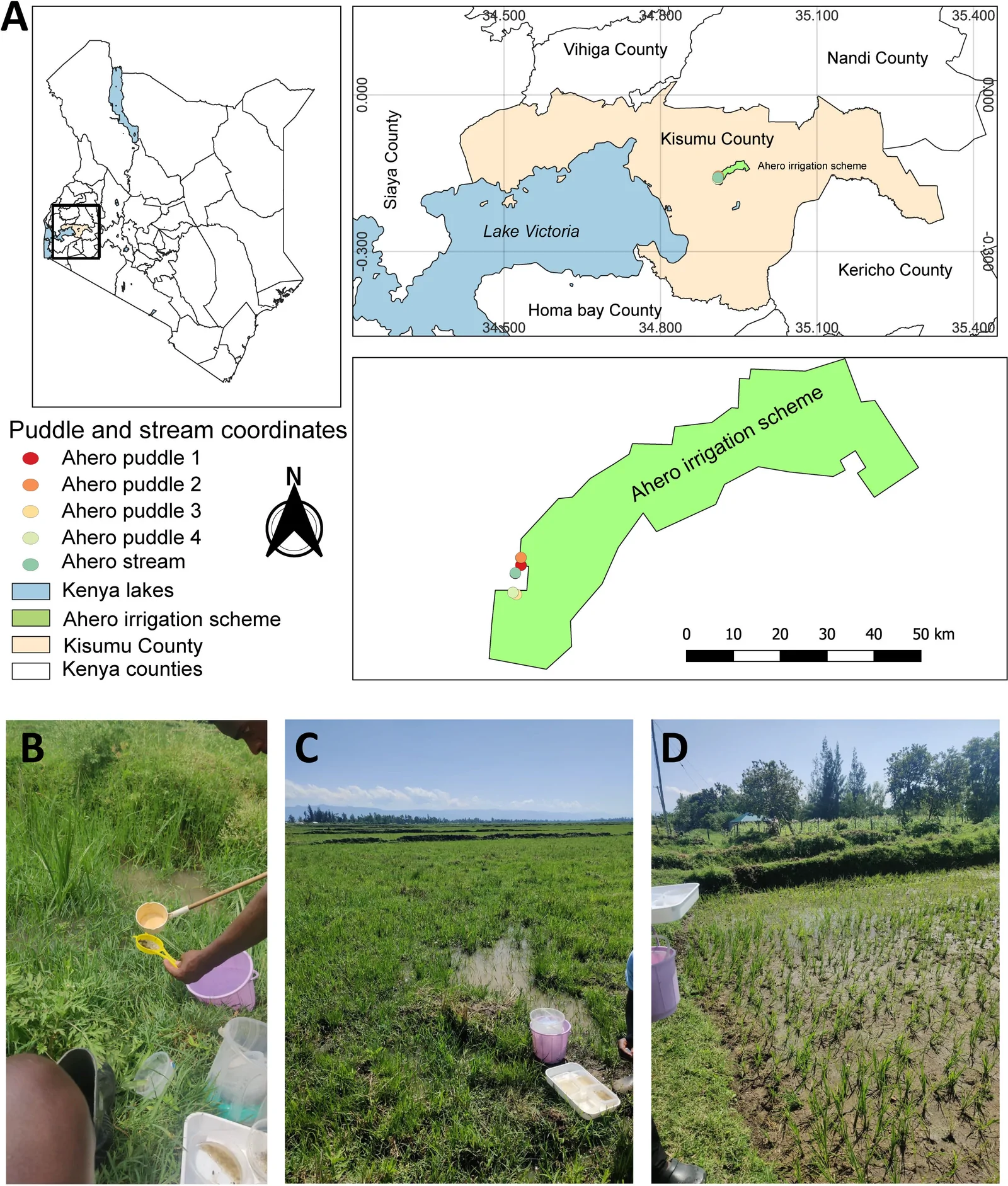
A map of Kenya showing the sampling sites in Kisumu County. (**A**) Shows the locations of the aquatic habitats within the Ahero irrigation scheme in Kisumu County, where aquatic macrofauna, anopheline, and non-anopheline larvae and pupae were sampled. The map was created using the open-source software QGIS v.3.14.16. (**B–D**) Representative images of the temporary puddles where collections were done. (**B**) The Ahero stream, (**C) **puddle 1, and (**D**) puddle 4, which are close to homesteads (in the background) where *Microsporidia M*B-infected mosquitoes have been collected.

This study was conducted in the Ahero irrigation scheme located in Kisumu County in the western part of Kenya (Fig. 1A). A purposive sampling strategy was used to select aquatic habitats that support *Anopheles* larval development and are located within villages where *Microsporidia MB*-infected adult *An. arabiensis* have been previously reported (10). Sampling focused on both mosquito immature stages and the associated aquatic community to characterize organisms potentially involved in the ecology and transmission of *Microsporidia MB* in aquatic environments. The mosquito larvae and pupae, as well as aquatic macrofauna, were collected from four temporary puddles and a stream (Fig. 1B through D) within the Ahero irrigation scheme from GPS coordinates: Puddle 1 (−0.15693624, 34.91088), Puddle 2 (−0.15577897, 34.91088), Puddle 3 (−0.16133336, 34.91019), Puddle 4 (−0.16103631, 34.90968), and stream (−0.15813829, 34.91003) between March and June 2025. The sampling period was selected to capture part of the seasonal variation in *Microsporidia MB* prevalence previously reported in Ahero, spanning months of relatively high prevalence (February–April) and subsequent decline (April–June) (10), thereby allowing assessment of potential hosts across a range of naturally occurring transmission conditions. Five field collections were conducted between March and June 2025. Pairwise distances among sampled puddles and streams ranged from 65.6 to 619.1 m, representing spatially distinct aquatic habitats within the irrigation landscape. Collections were made using a standardized dip-sampling protocol with a 350 mL mosquito dipper supported by a metal frame. Sampling was conducted systematically along accessible habitat margins at each site to ensure consistency in collection effort. Specimens were preserved in isopropanol immediately to maintain tissue integrity and transported to the ICIPE laboratories in Nairobi for morphological identification and molecular screening.

### Taxonomic classification of the aquatic macrofauna

### iNaturalist taxonomic classification

High-quality pictures of the aquatic macrofauna collected in the anopheline-associated habitats were taken using a stereomicroscope (ZEISS Stemi 508, ZEN Lite V.3.4., Oberkochen, Germany, identification number 195-044085) fitted with the Axiocam Erc5s camera. The images, together with the geographic coordinates of the collection sites, were uploaded to a dedicated project on the citizen-science platform iNaturalist ( https://www.inaturalist.org/projects/aquatic-macrofauna-collected-in-four-puddles-and-a-stream-in-ahero-irrigation-scheme-western-kenya). iNaturalist is a global community-based biodiversity platform that combines automated image-recognition algorithms with expert and community input to facilitate taxonomic identification of organisms and document their occurrence (24). This platform was used as the initial step in taxonomic identification of aquatic macrofauna. Taxonomic assignments were accepted when there was strong agreement among at least two experienced identifiers whose profiles indicated expertise in entomology and a substantial history of taxonomic identifications on the platform. The resulting identifications were used to guide specimen cataloging and subsequent confirmatory molecular analyses. Genomic DNA was subsequently extracted from individual organisms, as described below, to molecularly validate the taxonomic identifications obtained through iNaturalist.

### Genomic DNA extraction

Total DNA was isolated from the field-collected aquatic macrofauna using the protein precipitation method (10). Briefly, the samples were homogenized in individual 1.5 mL microcentrifuge tubes containing three sterile 0.5 mm beads and 50 µL of PBS using a BioSpec Mini Beadbeater for 1 min. The cell lysis buffer (300 µL) (5 mM EDTA, 0.5% SDS, 10 mM Tris (pH 8.0)) was added to the tubes, and the samples incubated at 65°C for 1.5 h. The protein precipitation solution (100 µL) (8 M Ammonium acetate, 1 mM EDTA) was added to the samples, after which they were lightly vortexed and placed at −4°C for 30 min. The samples were then centrifuged at 16,400 rpm for 15 min at 4°C. The supernatant was transferred to 1.5 microcentrifuge tubes containing 300 µL of molecular grade isopropanol (99.8%) and mixed by inversion 100 times. The samples were then centrifuged for an hour at 16,400 rpm at 4°C. The supernatant was poured off, and 300 µL of ice-cold 70% ethanol was added to the pellet left at the bottom of the tube. The tubes were inverted 50 times to wash the pellet and centrifuged for 30 min at 16,400 rpm at 4°C. The supernatant was poured off, and the pellets were air-dried. The nuclease-free PCR water (50 µL) was added to the dried pellet to elute the DNA, and the DNA was quantified using the ThermoScientific Nanodrop 2000c Spectrophotometer.

### Mitochondrial cytochrome c oxidase subunit I (COI) barcoding

Representative samples from each family and order classifications, as identified on iNaturalist, were barcoded using the COI gene to confirm the accuracy of the taxonomic identifications.

Extracted genomic DNA was used as a template for PCR amplification of the COI gene using gene-specific primers (Table 1). Briefly, a 22 µL PCR reaction consisting of 4 µL HOTFirepol R Blend Master Mix Ready-To-Load (Solis Biodyne, Estonia, mix composition: 7.5 mM magnesium chloride, 2 mM of each dNTP, HOT FIREPol R DNA polymerase), 1 µL each of the forward and reverse primers (10 pmol/µL), 2 µL of the DNA template, and 14 µL nuclease-free water was used. The PCR thermocycling conditions were as follows: initial denaturation at 95°C for 4 min, 35 cycles of repeated denaturation at 94°C for 30 s, annealing at 50°C for 30 s, extension at 72°C for 45 s, and final elongation at 72°C for 10 min. Agarose gel electrophoresis was done (120 V for 30 min) to resolve 5 µL of the PCR amplicons, with an expected band size of approximately 660 bp. The remaining PCR amplicons were purified using Exosap II (New England Biolabs, UK) according to the manufacturer’s instructions and subsequently submitted to Macrogen Europe (Amsterdam, The Netherlands) for Sanger sequencing.

**TABLE 1:** Primers for PCR amplification

Primer name	5′–3′ sequence	Primer orientation	Target	Fragment size (bp)	Refs.
MB18S F	CGCCGGCCGTGAAAAATTTA	Forward	*Microsporidia MB*	467	(10)
MB18S R	CCTTGGACGTGGGAGCTATC	Reverse			
Zianni primers	GTGAAGCTTGGTGCGTGCT	Forward	*An. arabiensis* and *An. gambiae*	165	(25)
	GCACGCCGACAAGCTCA	Reverse			
LCO1490-F	GGTCAACAAATCATAAAGATATTGG	Forward	COI gene	710	(26)
HCO2198-R	TAAACTTCAGGGTGACCAAAAAATCA	Reverse			

### Chromatogram analysis

The forward and reverse chromatograms of each representative of the aquatic macrofauna were trimmed, edited, and aligned using Geneious Prime software version 2024.0.7 to generate a consensus sequence (27). This consensus sequence was then queried against annotated sequences in the GenBank of NCBI database (https://www.ncbi.nlm.nih.gov/) using the BLAST nucleotide search tool (28) to confirm the taxonomic identity of the aquatic macrofauna. Sequences that yielded hits below 90% similarity were queried against annotated sequences in the BOLD database for confirmation of the taxonomic identifications (29). The family-level taxonomic identity was taken. A comparison was subsequently done between the taxonomic identifications provided by iNaturalist and COI barcoding to determine the accuracy of the iNaturalist identifications. All COI sequences generated in this study were deposited in the GenBank nucleotide database under accession numbers PZ532333–PZ532348.

### Speciation of mosquito larvae and pupae

The mosquito larvae were morphologically identified using published keys (30) to distinguish anopheline from non-anopheline larvae. Genomic DNA was extracted from the immature stages of the mosquito samples using the method described above. Species-specific PCR primers (Table 1) targeting diagnostic ribosomal DNA sequences were used to distinguish anopheline from non-anopheline larvae and pupae using an established high-resolution melt analysis protocol (25) with minor modifications. Briefly, an 11 µL PCR reaction consisting of 2 µL HOTFirepol Evagreen HRM Mix (no ROX) (Solis Biodyne, Estonia, mix composition: 5× EvaGreen HRM buffer, 12.5 mM magnesium chloride, dNTPs, HOT FIREPol DNA polymerase, EvaGreen dye, and BSA), 0.5 µL each of the forward and reverse primers (10 pmol/µL), 2 µL of the DNA template, and 6 µL nuclease-free water was used. The PCR thermocycling conditions were as follows: initial denaturation at 95°C for 15 min, 35 cycles of repeated denaturation at 95°C for 30 s, annealing at 58°C for 30 s, and extension at 72°C for 30 s, and final elongation at 72°C for 5 min. Finally, a 72–95°C melt with a 0.3°C increase per second was done to melt the DNA double-strands. The run was performed on the MIC qPCR cycler, and high-resolution melt (HRM) analysis was done to determine whether the mosquito samples were *An. arabiensis*, *An. gambiae*, or non-anopheline species. Sample melt curves of unknown samples were compared with those of reference positive controls derived from genetically characterized, insectary-maintained *An. arabiensis* and *An. gambiae* colonies. Samples whose melt curves exhibited peak melting temperatures corresponding to the *An. arabiensis* reference control were classified as *An. arabiensis*, whereas those matching the *An. gambiae* reference control were classified as *An. gambiae*. Samples exhibiting alternative melt curve profiles were classified as non-anopheline mosquitoes.

### Screening for *Microsporidia MB*

*Microsporidia MB* was screened as previously described (10). Briefly, DNA extracted from mosquito larvae, pupae, and aquatic macrofauna was subjected to PCR amplification using *Microsporidia MB*-specific primers targeting the 18S rRNA gene (MB18S; Table 1) to determine the presence of the symbiont. PCR reactions were performed in a total volume of 11 µL containing 2 µL HOT FIREPol Blend Master Mix Ready-To-Load (Solis BioDyne, Estonia; final reaction composition: 7.5 mM MgCl_2_, 2 mM of each dNTP, and HOT FIREPol DNA polymerase), 0.5 µL each of the forward and reverse primers (10 pmol/µL), 2 µL template DNA, and 6 µL nuclease-free water. Amplifications were performed under the following cycling conditions: initial denaturation at 95°C for 15 min, 35 cycles of repeated denaturation at 95°C for 30 seconds, annealing at 62°C for 30 s, and extension at 72°C for 30 s, and final elongation at 72°C for 5 min (10). PCR amplicons were resolved by electrophoresis on a 1.5% agarose gel at 120 V for 30 min and visualized under UV illumination. Samples producing the expected *Microsporidia MB* amplicon of approximately 467 bp were scored as positive.

## RESULTS

### Community structure of aquatic macrofauna in mosquito breeding habitats

**Fig 2:**
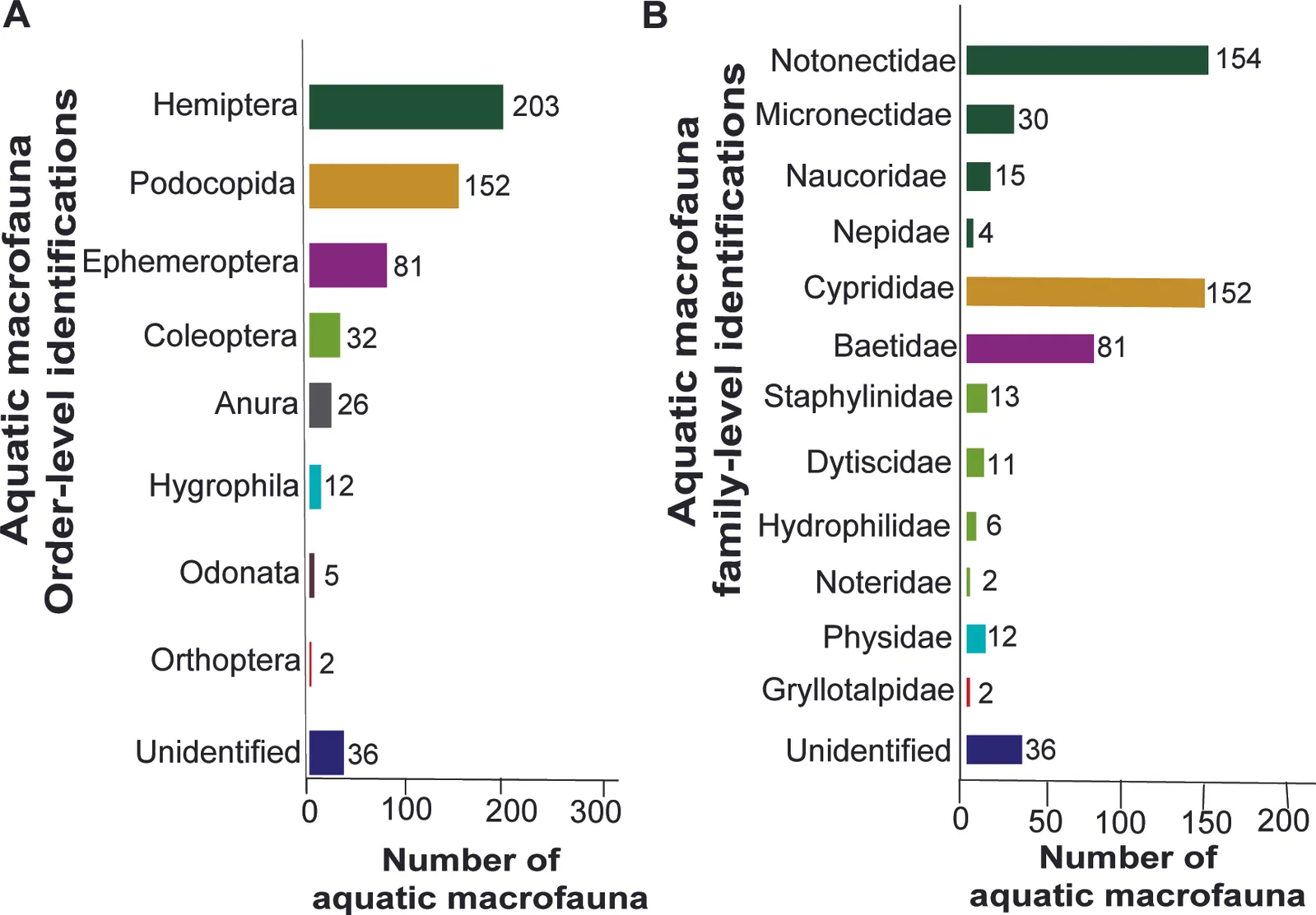
A summary of the taxonomic identifications of the collected aquatic macrofauna obtained from the combination of iNaturalist and COI barcoding. (**A**) The summary of the order-level identifications of the collected aquatic macrofauna from combining iNaturalist and COI barcoding. (**B**) The distribution of the family-level identifications obtained of the collected macrofauna, color-coded to represent their order-level taxa. The unidentified organisms did not have a consensus on their identifications on iNaturalist and were not barcoded using the COI gene; therefore, their identities are unknown.

A total of 549 aquatic macrofauna were collected from anopheline-associated breeding habitats. These included aquatic invertebrates (Classes Insecta, Gastropoda, and Ostracoda) and vertebrates (Class Amphibia). Four hundred ninety-eight organisms were taxonomically identified on iNaturalist to various taxonomic levels (Subphylum, Class, Order, Suborder, Infraorder, Superfamily, Family, Subfamily, and Genus), while 51 organisms were unidentified due to a lack of consensus among identifiers and a lack of identifications from the community. To assess the accuracy of taxonomic assignments generated using iNaturalist, representative specimens from the different taxonomic groups were subjected to COI DNA barcoding. Comparison of iNaturalist identifications with COI-based classifications showed complete concordance (100%) at both the class and order levels, indicating that the platform provides reliable higher-level taxonomic assignments. In contrast, concordance at the family level was lower (68.18%), suggesting reduced accuracy at finer taxonomic resolution. This discrepancy may reflect the fact that many observations had not attained research-grade status on iNaturalist, potentially increasing the likelihood of misclassification at lower taxonomic ranks. Nevertheless, integrating iNaturalist-based identifications with COI barcoding provided a robust approach for taxonomic assignment and helped resolve potential misidentifications within the data set. In addition, COI barcoding enabled the taxonomic identification of 15 of the 51 previously unidentified specimens to the family level, including members of the Physidae, Hydrophilidae, and Dytiscidae families. Overall, the combined use of iNaturalist and COI barcoding resulted in 482 aquatic macrofauna specimens being classified to the family level, 26 to the order level, and 5 to the suborder level. Thirty-six specimens remained unidentified because they either received no taxonomic assignment or lacked consensus among iNaturalist identifiers and were not subjected to COI sequencing (Fig. 2).

The identified aquatic macrofauna belonged to the Class Insecta, Orders Hemiptera (true bugs) (36.97%), Coleoptera (beetles) (5.83%), Orthoptera (0.36%), Ephemeroptera (mayflies) (14.75%), and Odonata (0.91%); Class Ostracoda, Order Podocopida (shrimp) (27.69%); Class Gastropoda, superorder Hygrophila (2.19%), and Class Amphibia, order Anura (tadpoles) (4.74%). The Order Hemiptera was the most dominant in the collection (36.97%), consisting of the families Naucoridae, Micronectidae, Nepidae, and Notonectidae. Notonectidae was the most dominant family in the Hemiptera order and the overall collection, making up 75.86% of the Hemiptera order and 28.05% of the overall collection (Table 2).

**TABLE 2:** The taxonomic distribution of the aquatic macrofauna collected during the study^*a*^

	Order	Family	*n*	% of Total
Insecta	Hemiptera (true bugs)	Naucoridae (creeping water bugs)	15	2.73
		Micronectidae (pygmy water boatmen)	30	5.46
				
		Nepidae (water scorpions)	4	0.73
		Notonectidae (backswimmers)	154	28.05
	Coleoptera (beetles)	Staphylinidae (rove beetles)	13	2.37
		Noteridae (burrowing water beetles)	2	0.36
		Hydrophilidae (water scavenger beetles)	6	1.09
		Dytiscidae (predaceous diving beetles)	11	2.00
	Orthoptera	Gryllotalpidae (mole crickets)	2	0.36
	Ephemeroptera	Baetidae (mayflies)	81	14.75
	Odonata	Unknown	5	0.91
Amphibia	Anura (frogs and toads)	Unknown	26	4.74
Ostracoda	Podocopida	Cyprididae (shrimp)	152	27.69
Gastropoda	Hygrophila	Physidae (Pouch snail)	12	2.19
Unidentified	Unknown	Unknown	36	6.56
		Total	549	100

^*a*^ n = Number of organisms collected; % of Total = percentage of each organism relative to the total number of organisms collected.

### Species composition of immature mosquito stages

A total of 544 mosquito larvae and 116 mosquito pupae were collected from the four puddles. Of these, 438 larvae and 109 pupae were identified as non-anopheline, and 106 larvae and 7 pupae were anophelines. Species identification revealed that 105 larvae and all 7 pupae were *An. arabiensis,* whereas only a single larva was identified as *An. gambiae* (Table 3).

**TABLE 3:** The distribution of collected aquatic macrofauna, larvae and pupae, across the sampled puddles in the study

Organisms collected		Field collection site				
	Puddle 1	Puddle 2	Puddle 3	Puddle 4	Stream	Total
Non-anopheline larvae	308	77	23	30	0	438
Non-anopheline pupae	107	2	0	0	0	109
Aquatic macrofauna	172	130	91	3	153	549
Anopheline larvae	0	20	74	12	0	106
Anopheline pupae	0	7	0	0	0	7

### Prevalence of *Microsporidia MB* in aquatic macrofauna and immature mosquito stages

**Fig 3:**
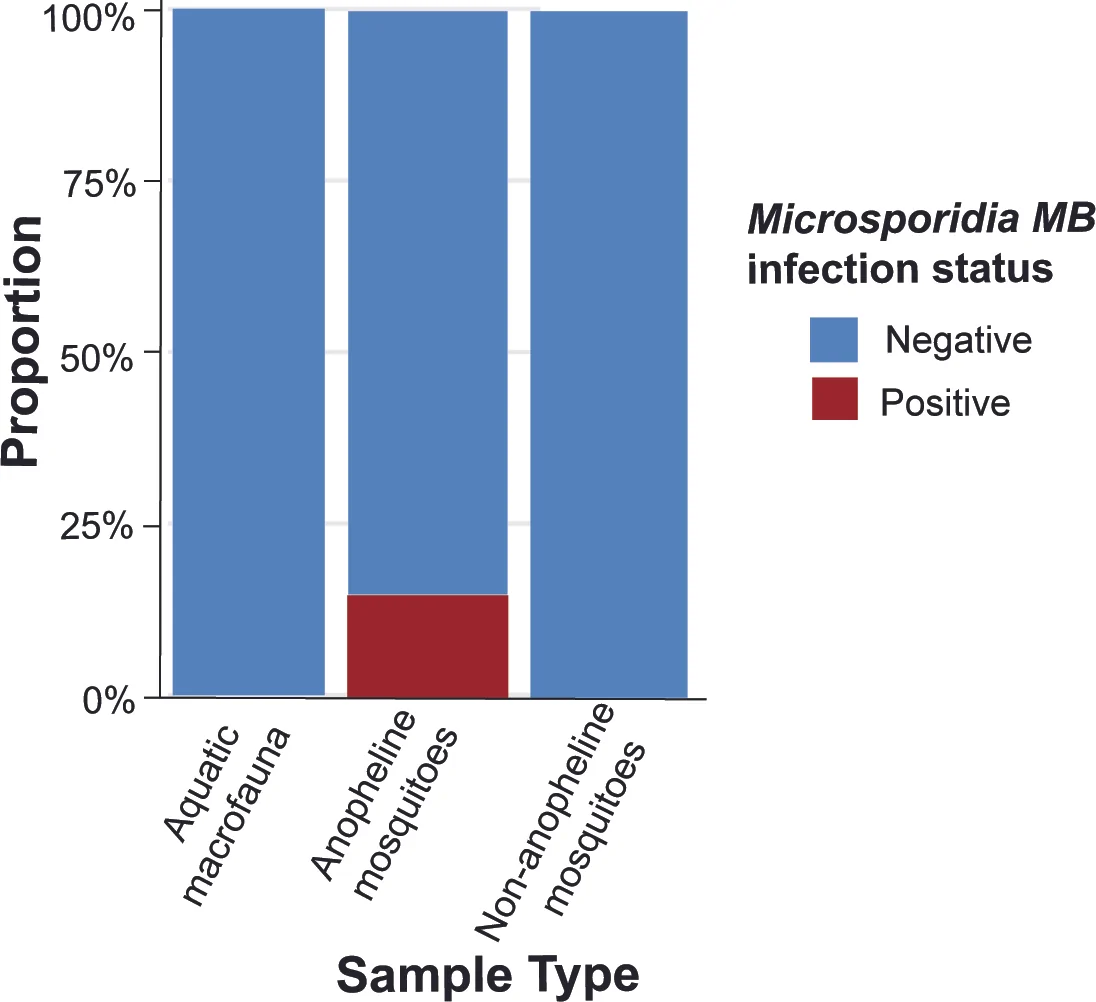
: *Microsporidia MB* infections in breeding sites: barplot showing proportion of *Microsporidia MB* infections in the aquatic macrofauna, anopheline mosquitoes, and nonanopheline mosquitoes collected from the temporal puddles. *Microsporidia MB* was detected in anopheline mosquitoes only.

All the collected aquatic macrofauna, mosquito larvae, and pupae were screened for *Microsporidia MB. Microsporidia MB* was detected exclusively in *Anopheles arabiensis* larvae, while all non-anopheline larvae, pupae, and aquatic macrofauna tested negative. In total, *Microsporidia MB*-positive larvae constituted 15.04% of the *An. arabiensis* larval population collected. Despite co-occurring in the same aquatic habitats, no infections were detected in non-anopheline taxa or aquatic macrofauna (Fig. 3).

## DISCUSSION

This study provides new insights into the ecology and host range of *Microsporidia MB*, an endosymbiont of anopheles mosquitoes with considerable potential as a malaria transmission-blocking agent. Through comprehensive molecular screening of a taxonomically diverse assemblage of aquatic macrofauna co-occurring within anopheline larval habitats, we found that *Microsporidia MB* was exclusively associated with anopheline mosquitoes. Specifically, the symbiont was detected only in *Anopheles arabiensis* larvae, where it occurred at a prevalence of 15.04%, and was absent from all non-anopheline mosquito species, aquatic macrofauna, and pupal samples based on our molecular screening approach. These findings suggest a high degree of host specificity and provide no evidence for a broader aquatic host range within the communities surveyed.

The prevalence observed in *An. arabiensis* larvae is consistent with the generally low prevalence of *Microsporidia MB* reported in field-collected mosquito populations, where infection rates typically range from 0% to 9% (10, 11, 19–21). The relatively low prevalence in larvae, coupled with the absence of detectable infections in pupae, may reflect the imperfect vertical transmission previously reported for this symbiont (13). In addition, metamorphosis in holometabolous insects involves extensive tissue remodeling, apoptosis, and histolysis, processes that can alter the distribution and abundance of endosymbionts within the host (31). It is therefore plausible that *Microsporidia MB* undergoes spatial reorganization or a transient reduction in density during pupation, resulting in infection levels below the detection threshold of our molecular assays. Further investigations tracking infection dynamics across developmental stages would help determine whether the absence of detectable infections in pupae reflects a true loss of the symbiont or a temporary decline in abundance during metamorphosis.

The absence of *Microsporidia MB* across a broad taxonomic range of aquatic invertebrates and vertebrates in this study is particularly striking given that many of these taxa interact closely with *Anopheles* larvae as predators, competitors, or habitat associates. In other microsporidian transmission systems, predators are known to play a role in the propagation of microsporidians in their target hosts. For instance, the predatory beetle, *Pterostichus gebleri*, aids the propagation of microsporidian *Paranosema locustae* in grasshopper populations (32). Once the predator consumes infected grasshoppers, it releases viable spores in its feces, which remain infectious to grasshoppers for up to 2 years (32). Some microsporidians have also incorporated mosquito larval predators, such as copepods, as their intermediate hosts in their life cycle. The *Amblyospora albifasciati* microsporidian, for example, uses the copepod *Mesocyclops annulatus*, voracious predators of first instar larvae (33), as its intermediate host (34) to facilitate horizontal transmission to larvae. Similarly, the microsporidian *Hyalinocosta chapmani*, which heavily relies on horizontal transmission, uses copepods as a reservoir to continually release infectious meiospores into the environment to sustain its transmission mechanisms (35). In this study, we found hemipterans (Notonectidae and Micronectidae) as common inhabitants of *Anopheles* breeding habitats, making up 36.97% of total collections. Hemipterans have been identified as voracious predators of mosquito larvae based on laboratory and semi-field predation assays (22, 36). Additionally, molecular gut-content analyses from natural breeding habitats (37) and long-term field surveys (7, 23, 38, 39) confirm that hemipteran predators frequently consume *Anopheles* larvae. In addition to hemipteran predators, 5.83% and 0.91% of collected organisms were diving beetles (Coleoptera: Dytiscidae and Hydrophilidae) and dragonflies (order Odonata) that are also active predators of *Anopheles* larvae, as demonstrated by laboratory and semi-field assays and field predator surveys (33). We also recorded the presence of competitors or co-occurring taxa of *Anopheles* mosquitoes, such as hemipterans Gryllotalpidae (0.36%) and Baetidae (14.75%), crustaceans (Cyprididae; 27.69%), snails (Physidae; 2.19%), and frogs and toads (Anura; 4.74%). The consistent presence of these predators/competitors and co-occurring taxa in *Anopheles* breeding sites supports prior evidence of their close interactions with aquatic stages of *Anopheles* mosquitoes. However, the absence of *Microsporidia MB* infections using our molecular screening approach in these taxa suggests that these organisms are unlikely intermediate or secondary hosts in the life cycle of this symbiont. While the absence of detection does not preclude the possibility of low-intensity, transient, or non-replicative associations below the detection threshold of our molecular screening approach, the findings suggest that the predatory, competing, and co-occurring organisms investigated are unlikely to represent major hosts supporting substantial *Microsporidia MB* amplification or persistence. Instead, the consistent detection of high-intensity infections in anopheline mosquitoes points to a strong host association and indicates that, if alternative hosts exist, they likely occur outside the taxa surveyed here or play a limited role in the ecology of the symbiont. Since the symbiont was detected in anopheline mosquitoes collected from all sampled habitats, it is also unlikely that environmental variation could have obscured detection in non-target aquatic taxa based on previous reports indicating that factors such as water quality affect *Microsporidia MB* prevalence in larval mosquito populations (21). Nevertheless, future studies incorporating habitat-level environmental measurements may provide further insight into additional ecological drivers of *Microsporidia MB* transmission.

These results support the specificity of *Microsporidia MB* to *Anopheles* mosquitoes observed in a previous study that reported the absence of *Microsporidia MB* infections in non-anopheline species and crustaceans in the genera *Mesocyclops*, *Macrocyclops*, and *Daphnia* that were inhabiting the same habitats as *An. arabiensis* (11). This is not uncommon since many microsporidians demonstrate host specificity by typically restricting themselves to a single host or a group of affiliated hosts (40). While we cannot conclusively state that *Microsporidia MB* lacks an alternative host as per the present study, which mainly focused on aquatic macrofauna due to their ecological overlap with mosquito larvae and their established role as hosts in other microsporidian systems, future studies should investigate meiofaunal and microfaunal communities that may harbor low-abundance or cryptic associations with *Microsporidia MB*. Furthermore, future experimental exposure and predator-feeding assays will be important for determining whether *Microsporidia MB* spores can persist within potential aquatic secondary hosts following ingestion and whether such interactions contribute to the environmental ecology of the symbiont. Additionally, because this study focused on Kenya’s Ahero Irrigation Scheme, a broader surveillance of anopheline breeding habitats across diverse climatic regions is essential to better characterize the ecological niche and host distribution range of *Microsporidia MB* infections.

Microsporidians that use alternative hosts are often found to be heterosporous and sometimes use a combination of horizontal and vertical transmission (41). Although *Microsporidia MB* has been demonstrated to employ both vertical transmission to progeny and horizontal transmission through mating (10–14, 19), the formation of multiple spore types has not been demonstrated. A recent study found evidence of sporogony of *Microsporidia MB* in the oocyte, and evidence of spores within the laid eggs (15). However, this study did not reveal the existence of different forms of *Microsporidia MB* spores (15). Further investigations should be done to determine if *Microsporidia MB* produces other forms of spores, especially within the natural breeding habitats of *Anopheles* mosquitoes. Interestingly, the analysis of the *Microsporidia MB* genome reported the presence of meiosis-related genes, which may imply a possible form of sexual reproduction in the symbiont (42). Meiosis is an integral process in the formation of meiospores (43), which are infectious to alternative hosts or mosquito larval hosts, and are largely produced by microsporidians that include a secondary host in their life cycles.

### Conclusion

Our findings support the hypothesis that *Microsporidia MB* maintains a highly restricted host range and is strongly associated with anopheline mosquitoes. Although low-intensity or transient associations with other aquatic organisms cannot be entirely excluded, the absence of detectable infections across a diverse assemblage of co-occurring aquatic taxa suggests that the investigated species are unlikely to play a major role in the ecology or transmission of the symbiont. This apparent host specificity has important implications for the development of *Microsporidia MB* as a symbiont-based malaria intervention, as it reduces concerns regarding unintended spread to non-target organisms while supporting environmental containment (44). Together with its demonstrated malaria-blocking phenotype and efficient transmission within mosquito populations, these characteristics strengthen the case for *Microsporidia MB* as a targeted and sustainable complementary tool for malaria control. More broadly, our findings support the development of *Microsporidia MB* as a vector-control strategy to complement existing interventions in the face of persistent malaria transmission and increasing insecticide resistance.

## Data Availability

Data on the aquatic macrofauna and immature stages of mosquitoes collected from Anopheles breeding habitats are available at Figshare Digital Repository at 10.6084/m9.figshare.31398042. COI sequences used to confirm taxonomic identifications have been deposited into the GenBank (accession numbers PZ532333–PZ532348).
